# The Psychosocial Work Environment and Perceived Stress among Seniors with Physically Demanding Jobs: The SeniorWorkingLife Study

**DOI:** 10.3390/ijerph18147437

**Published:** 2021-07-12

**Authors:** Jonas Vinstrup, Annette Meng, Emil Sundstrup, Lars L. Andersen

**Affiliations:** 1 National Research Centre for the Working Environment, 2100 Copenhagen, Denmark; ame@nfa.dk (A.M.); ESU@nfa.dk (E.S.); LLA@nfa.dk (L.L.A.); 2 Sport Sciences, Department of Health Science and Technology, Aalborg University, 9220 Aalborg, Denmark

**Keywords:** psychological stress, workload, physical exertion, blue-collar workers, Cohen

## Abstract

Background: Poor psychosocial work conditions are known to foster negative health consequences. While the existing literature on this topic focus mainly on white-collar workers, the influence of different aspects of the psychosocial work environment in physically demanding jobs remain understudied. Likewise, senior workers represent a population of the workforce at increased risk of adverse health outcomes and premature exit from the labour market. This study investigates the association between psychosocial work factors and perceived stress among the senior work force. Methods: Utilizing cross-sectional findings, this study reports associations between psychosocial factors (organizational justice, cooperation and collegial support, decision latitude, clarity of tasks, and quality of leadership) and the outcome of perceived stress quantified by Cohen’s Perceived Stress Scale (CPSS). Currently employed senior workers with physically demanding jobs were included in the analyses (n = 3386). Associations were modeled using general linear models with weights to make the estimates representative. Results: For all individually adjusted psychosocial variables, the category of “good” was consistently associated with lower stress scores compared to the categories of both “moderate” and “poor” (all *p* < 0.0001). Likewise, in the mutually adjusted analysis, the category of “good” was statistically different from “poor” for all included variables, while the category of “moderate” remained different from “poor” for “clarity of tasks”, “cooperation and collegial support”, and “decision latitude”. Conclusions: Among senior workers with physically demanding jobs, poor ratings of organizational factors related to the psychosocial work environment are consistently associated with high stress scores. Blue-collar occupations focusing primarily on physical risk factors are recommended to increase awareness on psychosocial aspects that may be relevant to the local work environment.

## 1. Introduction

Since the first scientific article with the term “psychosocial work environment” embedded in the title was published in 1982 [1], this field of research has experienced exponential growth within academia and public health alike [2]. Likewise, the importance of psychosocial aspects within the local work environment has increasingly been recognized in the literature, giving rise to the notion that the physical demands and conditions are far from the be-all-end-all of a long, healthy, and productive work-life [3,4,5,6,7].

Among middle-aged workers, factors related to the psychosocial work environment have been shown to predict work exit by age 50. Specifically, low decision latitude, poor collegial support, and job insecurity double the risk of early work exit [8], whereas favorable psychosocial working conditions seem to significantly reduce this risk and may even facilitate working beyond pension age [9]. Recent results from a 20-year follow-up analysis of the Whitehall II study showed that high skill discretion and work-related social support effectively diminish the risk of premature exit from the labor market [10]. Likewise, the inherent quality of a good psychosocial work environment is not only essential in retaining workers but has also shown to be of vital importance for the employee returning from sick leave. In short, even when adjusting for health complaints as well as current and expected future work ability, decision latitude and job promotion opportunities continue to predict return to work [11].

However, in the attempt to quantify the deleterious consequences of a poor psychosocial work environment, it is inherently difficult to separate these from the effects of stress. Alas, it is likely that psychosocial stress serves as a potent intermediate between a poor work environment and negative health outcomes [3,5,12,13,14,15,16]. For example, subjective ratings of the work environment have been found to correlate with biomarkers of stress [15], and a recent systematic review found psychosocial work stressors to be associated with an increased risk of all-cause mortality among the general working population [3]. Thus, differentiating between potential predictors and—perhaps more importantly—identifying specific aspects of the work environment associated with psychosocial stress seem of utmost importance and would enable targeted health strategies at the workplace. Additionally, while the majority of stress-related research has focused on white-collar workers, it is likely that the psychosocial work environment of their blue-collared counterparts have been disproportionally neglected [17]. Lastly, in addition to being more vulnerable to work-related adverse health outcomes [18], senior workers constitute a population of the workforce experiencing increased risk of premature exit from the labour market, of which namely seniors with physically demanding work have less opportunities in the workplace for supporting a prolonged work life [19].

Therefore, the present study sought to investigate associations between individual aspects of the psychosocial work environment and stress among seniors with physically demanding jobs. Since these job groups traditionally focus mainly on levels of physical exposure during work, the aim of this study was to emphasize aspects of the psychosocial work environment related to perceived stress.

## 2. Methods

### 2.1. Study Design and Population

Utilizing a cross-sectional study design, this study reports associations between occupational psychosocial factors and perceived stress. A large-scale questionnaire survey was dispatched in July 2018 to 30,000 Danes above the age of 50 (18,000 employed, 7000 unemployed, 3000 on voluntary early retirement, 2000 on disability pension), drawn as a probability sample at Statistics Denmark, and merged with national registers through individual social security numbers [20]. Publications from this research program can be found in PubMed using the term “SeniorWorkingLife”.

Initiated in 2018, the project investigates push and stay mechanisms among older workers and aspires to repeat the survey every 2–3 years. In the present analyses, 12,173 currently employed senior workers replied to the question about physical work demands (four categories from “sedentary” to “physically strenuous”). For the present article, we included only the two (self-reported) categories related to physically demanding jobs (i.e., “primarily walking/standing work with a lot of lifting or carrying” or “primarily physically strenuous heavy/fast work”) (n = 3566). Finally, we excluded those with depression, leaving a total sample size of 3386. The 10 largest job groups, in descending order, were: (1) nurse’s aides, (2) machine operators, (3) construction workers, (4) cleaners, (5) farmers and gardeners, (6) bricklayers and plumbers, (7) welders, (8) building and cleaning supervisors, (9) civil engineering labourers, and (10) carpenters and woodworkers.

The current study followed the STROBE recommendations for the reporting of cross-sectional studies [21] (ClinicalTrials Identifier: NCT03634410).

### 2.2. Predictors

Inspired by the Copenhagen Psychosocial Questionnaire (COPSOQ) [22] and adapted from the 2018 round of the Danish Work Environment Cohort Study (DWECS) [23], the present analyses include 12 individual items as predictors (Appendix A). These were rated on a 5-point Likert-scale (ranging from “never” to “always”) and subsequently merged into five categories related to different aspects of the psychosocial work environment. These were comprised of (1) organizational justice, (2) cooperation and collegial support, (3) decision latitude, (4) clarity of tasks, and (5) quality of leadership. For example, ratings to the questions “how often do you influence *how* you solve your work tasks?” and “how often do you influence *when* you solve your work tasks?”, represented in category 3, were averaged and converted into a 0–100 scale (i.e., poor; 0–50, moderate; 50–75, and good; 75–100). The weighted prevalence of each category is shown in Table 1. As different psychosocial factors are often related, we checked for multicollinearity (r > 0.70). The weighted Pearson correlation coefficient between the different factors ranged between 0.32–0.64, and therefore none of the factors were excluded from the mutually adjusted analysis.

### 2.3. Outcome

Cohen’s Perceived Stress Scale (CPSS-10; scale 0–40) was used as a continuous outcome measure representing psychosocial stress; i.e., used interchangeably with “perceived stress” as per the consensus that psychological, sociological, and environmental factors all contribute to the feeling of stress [24,25]. Consisting of 10 questions, with each item rated on a 5-point Likert scale ranging from “never” to “almost always”, the scale shows satisfactory validity, reliability, and internal consistency [26,27,28]. Likewise, the Danish consensus version of CPSS, used in the present study, exhibits cross-cultural adaptation and good psychometric properties [26].

### 2.4. Statistics

Associations were modelled using general linear models (Proc Glm, SAS) with CPSS-10 as the outcome (continuous variable, 0–40). The included psychosocial factors constituted the predictor variables. In model 1, each factor was entered separately (i.e., not mutually adjusted). Furthermore, this model was controlled for sex (from register), age (from register), highest attained education (from register), the type of physical work demands (from questionnaire), as well as lifestyle factors (from questionnaire: leisure time, physical activity, smoking habits, and body mass index). These covariates were chosen due to their potential (bi-directional) influence on psychosocial stress [29,30,31,32]. In model 2, all the psychosocial factors were entered simultaneously (i.e., mutually adjusted) and adjusted for the same control variables as model 1. In all analyses, model-assisted weights were applied to produce representative estimates. The weights were based on high-quality national registers at Statistics Denmark and took into account sex, age, occupational industry, highest completed education, family income, type, and origin [20]. Results were reported as least square means and 95% confidence intervals, as well as differences of least-square means and 95% confidence intervals.

## 3. Results

The current sample includes 3386 senior workers with physically demanding jobs, with Table 1 depicting demographics and weighted prevalence of the five included psychosocial variables related to the work environment.

Table 2 shows individually adjusted psychosocial variables and accompanying categories of “good” (reference), “moderate”, and “poor”. For all five psychosocial variables, the categories of “moderate” and “poor” were strongly associated with increased stress scores (all *p* < 0.0001). For example, for “organizational justice”, the categories of “good”, “moderate”, and “poor” corresponded to CPSS scores of 10.7 (95% CI 9.0–12.4), 12.2 (95% CI 10.5–13.9), and 14.4 (95% CI 12.8–16.1), respectively, with a similar pattern emerging from the remaining four variables.

Likewise, Table 3 shows mutually adjusted associations between the included psychosocial factors and stress scores: With the exception of the category of “moderate” for “organizational justice” and “quality of leadership” (*p* = 0.23 and *p* = 0.12, respectively), the categories of both “moderate” and “poor” remained associated with increases in stress scores for “cooperation and collegial support”, “decision latitude”, and “clarity of tasks” (all *p* ≤ 0.02).

## 4. Discussion

This study reports associations between ratings of pre-defined aspects of the psychosocial work environment and stress scores, showing clear differences in both the individually and mutually adjusted analyses. Of note, the category of “clarity of tasks” shows the largest differences, exhibiting mean CPSS scores of 11.2, 12.9, and 14.9 for the categories of “good”, “moderate”, and “poor”, respectively, while the category of “cooperation and collegial support” exhibits scores of 12.1, 13.0, and 13.9, respectively. While the directionality cannot be established with certainty, these results highlight a potential mediating effect of stress on health-related outcomes commonly attributed directly to the psychosocial work environment, as well as the importance of differentiating between its inherent components.

Of note, the present analyses were performed on senior workers with physically demanding jobs. Among this population of the workforce, the majority of research is customarily done in relation to the aspects of the physical work environment [33,34,35]. While this common differentiation between job groups and their (assumed) differences in work-related stressors likely stems from outdated societal notions, this study infers that psychosocial stress is also a highly relevant topic among workers with physically demanding jobs. In fact, when comparing occupations based on the International Standard Classification of Occupations (ISCO), we have recently shown that workers with physically demanding jobs not only exhibit increased odds of musculoskeletal pain compared with more sedentary jobs, they are also likely to experience at least the same degree of psychosocial stress [36]. Therefore, while the inherent components of demanding physical work—likely to foster negative health and productivity outcomes [33,37,38]—are not to be neglected, a growing body of evidence emphasizes the importance of including several aspects of the psychosocial work environment when informing policies [3,6,39]. In light of this, it is becoming increasingly clear that a broad array of very different organizational-, lifestyle-, and health-related factors relate to and indeed influence work ability [37,39,40,41]. Interestingly, while musculoskeletal pain—alone and in combination with high physical job demands—seems to be a strong predictor of poor work ability among the general working population [42] and senior workers alike [43,44], a range of psychosocial factors have been shown to modify these associations [39,45]. For example, in a nationally representative sample of middle-aged Finnish workers experiencing multi-site pain, 37% and 48% of men and women, respectively, report “good” (≥ 9, 0–10 scale) work ability [39]. Following this, by utilizing data from the same cohort in a 7-year follow-up, Haukka et al. identify several work and lifestyle factors that influence the outcome of long-term sickness absence, ranging from “possibility to adjust workday length” to “no problems with working community or mental stress” [45]. Therefore, these and other recent results indicate that several organizational psychosocial factors serve as both predictors and protective determinants of work ability [46,47].

Summarily, while it is perspicuous that a plurality of physical and psychosocial risk factors exists within the work environment, the ability of the latter to modify the former and hereby directly influence work ability even among pain-ridden workers needs to be emphasized. The present study provides additional insight into the prevalence of psychosocial stress among workers with physically demanding jobs, highlighting the importance of deferring the prevailing focus on physical risk factors among this group of workers.

### 4.1. Perspectives

Considering the job demands–resources model by Demerouti and Bakker—an overarching framework with application across a wide range of occupational settings [48,49,50,51]—work-related stress is characterized by a mismatch between the inherent work demands and the adaptive resources of the individual, ultimately resulting in the neuroplastic, physiological, and behavioral changes associated with prolonged psychosocial stress [52,53]. This notion of allostatic (over)load and consequential homeostatic disruption echoes through all aspects of research on human stress syndromes, highlighting the fact that the brain does not discriminate between origins of various stressors [53]. However, creating impactful changes in the local work environment in the attempt to decrease the prevalence of stress is not always feasible. Specifically, among senior workers with physically demanding jobs, it is likely that intrinsic components of the work limit the potential for change and that some aspects of the psychosocial work environment are therefore easier to attune than others. For example, while individual “decision latitude” might be difficult to influence among most blue-collar jobs with predominantly predetermined tasks, a more suitable target for improvement may, for example, be “cooperation and collegial support”, as it relates less to the specific task at hand.

Therefore, while it is clear that individual demands, resources, and coping behavior modulate the stress response, local working environment policies would benefit from effectively identifying both positive and negative characteristics inherent to the occupational setting that are modifiable and may influence the well-being of the worker [54].

### 4.2. Strengths and Limitations

Limitations include the inherent risks associated with questionnaire surveys, including recall, non-response, and, most notably, common-method bias [55,56,57]. In effect, the directionality of the presented associations cannot, per the cross-sectional design of the study, be established, as it is likely that workers experiencing stress are more prone to rate aspects of their psychosocial work environment as being poor. However, likely in a bi-directional manner, the associations between perceived stress and work-related psychosocial factors remain convincing.

While based on self-reporting, a noteworthy strength includes the use of the Danish version of CPSS-10 for assessing psychosocial stress [26,27], and by utilizing a probability sample merged with national registers through social security numbers, it is likely that the presented results adequately represent the population of Danish senior workers.

## 5. Conclusions

Poor ratings of the psychosocial work environment are associated with higher stress scores among senior workers with physically demanding jobs. Blue-collar occupations focusing primarily on physical risk factors are recommended to adapt current policies to emphasize the importance of psychosocial risk factors relevant to the local work environment. Specifically, highlighting aspects related to collegial support and clarity of tasks may especially be important among this group of workers. Finally, these results signify the importance of recognizing perceived stress as a potential mediator of the negative health consequences attributed to the psychosocial work environment.

## Figures and Tables

**Table 1:** Demographics and weighted prevalence of work-related psychosocial factors.

	n	Weighted Percentage (%)	Weighted Mean (SD)
Age (mean and 95% CI)	3386		56.6 (5.3)
BMI (mean and 95% CI)	3279		26.6 (5.5)
Females	1371	43.6	
Smoking (yes)	769	22.0	
**Organizational justice**			
Poor (0–50)	1362	42.0	
Moderate (50–75)	1340	40.6	
Good (75–100)	652	17.4	
**Cooperation and collegial support**			
Poor (0–50)	466	13.6	
Moderate (50–75)	1418	43.7	
Good (75–100)	1476	42.7	
**Decision latitude**			
Poor (0–50)	729	21.6	
Moderate (50–75)	1276	37.4	
Good (75–100)	1362	41.0	
**Clarity of tasks**			
Poor (0–50)	508	14.6	
Moderate (50–75)	1892	56.8	
Good (75–100)	948	28.6	
**Quality of leadership**			
Poor (0–50)	1188	36.8	
Moderate (50–75)	1235	36.2	
Good (75–100)	922	27.0	
**Level of physical activity during leisure time**			
Sedentary	608	18.2	
Light intensity exercise > 4 h/week	2008	61.3	
Moderate intensity exercise > 4 h/week	646	18.9	
High intensity exercise several times/week	46	1.6	

Values are presented as absolute numbers (n), weighted means with standard deviations (SDs), and weighted percentages (%).

**Table 2:** Model 1. Individually adjusted psychosocial variables and stress scores (0–40).

	Stress Score			
Psychosocial Variable	Mean	95% CI	Difference between Means (95% CI)	*p*-Value
**Organizational justice**				
Good	10.7	9.0–12.4		
Moderate	12.2	10.5–13.9	−1.5 (−2.0, −0.9)	<0.0001
Poor	14.4	12.8–16.1	−3.7 (−4.3, −3.2)	<0.0001
**Cooperation and collegial support**				
Good	11.2	9.5–12.9		
Moderate	13.4	11.7–15.1	−2.2 (−2.6, −1.9)	<0.0001
Poor	15.5	13.8–17.2	−4.3 (−4.9, −3.7)	<0.0001
**Decision latitude**				
Good	11.5	9.8–13.1		
Moderate	13.2	11.5–14.9	−1.7 (−2.2, −1.3)	<0.0001
Poor	15.2	13.5–16.9	−3.7 (−4.2, −3.2)	<0.0001
**Clarity of tasks**				
Good	10.2	8.5–11.9		
Moderate	12.7	11.1–14.4	−2.5 (−2.9, −2.1)	<0.0001
Poor	15.9	14.2–17.6	−5.7 (−6.3, −5.1)	<0.0001
**Quality of leadership**				
Good	10.8	9.1–12.5		
Moderate	12.4	10.7–14.1	−1.7 (−2.1, −1.2)	<0.0001
Poor	14.5	12.9–16.2	−3.8 (−4.3, −3.3)	<0.0001

Values are presented as means with 95% confidence intervals. Adjusted for age, sex, smoking, BMI, education, and level of physical activity during leisure time and work.

**Table 3:** Model 2. Mutually adjusted psychosocial variables and stress scores (0–40).

	Stress Score			
Psychosocial Variable	Mean	95% CI	Difference between Means (95% CI)	*p*-Value
**Organizational justice**				
Good	12.6	11.0–14.3		
Moderate	13.0	11.4–14.6	−0.3 (−0.9, 0.2)	0.23
Poor	13.4	11.8–15.0	−0.8 (−1.4, −0.1)	0.02
**Cooperation and collegial support**				
Good	12.1	10.5–13.8		
Moderate	13.0	11.3–14.6	−0.8 (−1.2, −0.4)	0.0003
Poor	13.9	12.2–15.5	−1.7 (−2.4, −1.1)	<0.0001
**Decision latitude**				
Good	12.3	10.7–14.0		
Moderate	12.9	11.3–14.5	−0.6 (−1.0, −0.1)	0.01
Poor	13.8	12.1–15.4	−1.4 (−2.0, −0.9)	<0.0001
**Clarity of tasks**				
Good	11.2	9.6–12.9		
Moderate	12.9	11.2–14.5	−1.6 (−2.1, −1.2)	<0.0001
Poor	14.9	13.2–16.5	−3.7 (−4.3, −3.0)	<0.0001
**Quality of leadership**				
Good	12.5	10.9–14.2		
Moderate	12.9	11.3–14.5	−0.4 (−0.9, 0.1)	0.12
Poor	13.5	11.9–15.2	−1.0 (−1.6, −0.4)	0.0008

Values are presented as means with 95% confidence intervals. Adjusted for age, sex, smoking, BMI, education, and level of physical activity during leisure time and work.

## Data Availability

The authors encourage collaboration and use of data by other researchers. Data are stored on the server of Statistics Denmark, and researchers interested in using the data for scientific purposes should contact project leader Prof. Lars L. Andersen, lla@nfa.dk.

## Appendix A. Individual Psychosocial Variables and Their Groupings

	**Individual Questions**	**Category**
1	How often are employees, who are affected by a given decision, heard?	Organizational justice
2	How often are all employees treated fairly at the workplace?	
3	How often do you and your colleagues help each other achieving the best possible result?	Cooperation and collegial support
4	At your workplace, how often are considerations taken towards employees with less energy (e.g., elderly or sick)?	
5	How often do you and your colleagues work together when problems arise that require solutions?	
6	How often do you have a say in how you complete your work tasks?	Decision latitude
7	How often do you have a say in when your complete your work tasks?	
8	How often do you receive the information, guidance and instructions you need in order to do your job?	Clarity of tasks
9	How often do you know exactly what your work tasks are?	
10	How often are you exposed to conflicting demands at work?	
11	How often is your work recognized and valued by management?	Quality of leadership
12	How often do you receive the help and support you need from your immediate manager?	
